# Chemical and Mechanical Characterization of Licorice Root and Palm Leaf Waste Incorporated into Poly(urethane-acrylate) (PUA)

**DOI:** 10.3390/molecules26247682

**Published:** 2021-12-19

**Authors:** Serena Gabrielli, Genny Pastore, Francesca Stella, Enrico Marcantoni, Fabrizio Sarasini, Jacopo Tirillò, Carlo Santulli

**Affiliations:** 1 ChIP Building, School of Science and Technology, Università degli Studi di Camerino, Via Madonna delle Carceri, 62032 Camerino, Italy; genny.pastore@unicam.it (G.P.); francesca.stella@unicam.it (F.S.); enrico.marcantoni@unicam.it (E.M.); 2 Department of Chemical Engineering Materials Environment, Sapienza-Università di Roma, Via Eudossiana 18, 00184 Roma, Italy; fabrizio.sarasini@uniroma1.it (F.S.); jacopo.tirillo@uniroma1.it (J.T.); 3 Geology Section, School of Science and Technology, Università degli Studi di Camerino, Via Gentile III da Varano 7, 62032 Camerino, Italy

**Keywords:** lignocellulosic materials, poly(urethane-acrylate), licorice root, oil palm leaf, mechanical properties, thermal properties

## Abstract

A poly(urethane-acrylate) polymer (PUA) was synthesized, and a sufficiently high molecular weight starting from urethane-acrylate oligomer (UAO) was obtained. PUA was then loaded with two types of powdered ligno-cellulosic waste, namely from licorice root and palm leaf, in amounts of 1, 5 and 10%, and the obtained composites were chemically and mechanically characterized. FTIR analysis of final PUA synthesized used for the composite production confirmed the new bonds formed during the polymerization process. The degradation temperatures of the two types of waste used were in line with what observed in most common natural fibers with an onset at 270 °C for licorice waste, and at 290 °C for palm leaf one. The former was more abundant in cellulose (44% vs. 12% lignin), whilst the latter was richer in lignin (30% vs. 26% cellulose). In the composites, only a limited reduction of degradation temperature was observed for palm leaf waste addition and some dispersion issues are observed for licorice root, leading to fluctuating results. Tensile performance of the composites indicates some reduction with respect to the pure polymer in terms of tensile strength, though stabilizing between data with 5 and 10% filler. In contrast, Shore A hardness of both composites slightly increases with higher filler content, while in stiffness-driven applications licorice-based composites showed potential due to an increase up to 50% compared to neat PUA. In general terms, the fracture surfaces tend to become rougher with filler introduction, which indicates the need for optimizing interfacial adhesion.

## 1. Introduction

A considerable amount of ligno-cellulosic waste is produced in several industries, spanning from the food sector to the manufacturing of furniture or to the disposal of vegetable refuse, after pruning or cutting operations. A possibility to re-use with an upcycling concept, therefore giving economical value to this process, can be its introduction as a filler in polymers. This process does normally require compatibilization to improve the wettability and adhesion of ligno-cellulose waste to the matrix [1]. Another significant factor to be accounted for is the geometry of the filler to be introduced. A number of studies on the introduction of fibrous agrowaste into polymer matrices are reported in [2], while in [3] literature on the combined presence of agrowaste with natural fibers in polymers is discussed. In particular, the improved physico-chemical performance of cellulose fibers when modified with poly(urethane-acrylate) (PUA) copolymers is well known [4]. In fact, during the last few decades, PUA has been widely used as tissue adhesives as well as UV coatings due to its unique properties as excellent abrasion resistance, adhesion to substrates, light stability, and weatherability [5].

Furthermore, it also results in a reduced wettability of the fibers [6], which suggests its potential as the matrix also for ligno-cellulosic waste.

As far as the selection of filler to be used is concerned, two categories can be clearly distinguished: the filler can either be a by-product in the food sector with limited value in itself, or else it might represent a non-marketable waste of a soundly established and widely diffuse practice, such as garden waste collection. An example of the former can be the use in polymers of almond [7,8], nut [9], or peanut powdered shells [10]. In contrast, as regards garden and pruning waste introduction into polymers, this has been rather regarded so far more as a possibility than practiced concretely [11]. On the other side, wood waste from a number of species has been considered as a source for lignin extraction, in particular aspen (*Populus tremula*) [12], spruce (*Picea abies*) or eucalyptus [13], or even oil palm biomass [14].

In this work, two types of biomasses, one from the first type, a by-product of the food industry, namely licorice root, and one of the second group, obtained from differentiated waste collection of garden waste, namely Canary date palm (*Phoenix canariensis*) leaf, have been compared as for their introduction in a poly(urethane-acrylate) (PUA) matrix. On the former, a previous study aimed at its introduction in epoxy offered some promising results [15]. However, PUA proved very suitable in general for its possible combination with lignocellulosic waste [16]. However, the attention towards diversifying the filler in this polymer has been limited so far. The availability of large amounts of differentiated biomass from a large number of species could open further opportunities for PUA in the fabrication of biocomposites. The novelty of the work is in the application of these two lignocellulosic waste fillers, as an example for other available fillers, into a poly(urethane-acrylate) (PUA) matrix, with the intent to characterize the capability of this polymer instead of the classical epoxy resins. PUA has been used in view of its capacity to offer effective coatings for lignocellulosic substrates for the possible quality of its interaction with these: this has been verified e.g., in the case of bamboo [17].

## 2. Materials and Methods

### 2.1. Materials

The reagents for the synthesis of poly(urethane-acrylate) (PUA), namely polyethylene glycol (PEG 400), isophorone diisocyanate (IPDI), the catalyst dibutylbis(lauroyloxy)tin and the end-capping agent 2-hydroxyethyl methacrylate (HEMA), were purchased from Merck Life Science (Milan, Italy) and used without further purification.

Two types of waste were introduced, licorice root waste following the extraction of licorice juice by ethanol, which was supplied by Amarelli-Fallani, Rossano Calabro, Italy, and palm tree waste, which was supplied by Ecoflora 2, Ardea, Italy, a firm that is in charge of collecting and exploiting pruning waste in the Rome area.

### 2.2. Preparation of PUA-Based Composites

**Figure 1:**
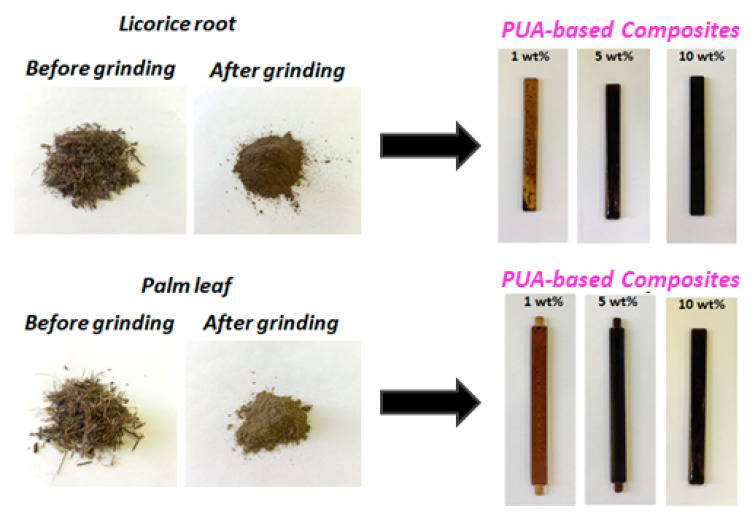
The two types of waste and PUA-based composites with different amounts of waste filler.

Poly(urethane-acrylate) obtained with the optimized procedure was filled with licorice root and palm leaf waste in powder form (Figure 1), previously dried in an oven at 80 °C for 24 h and milled. Three different concentrations of filler were considered: 1, 5 and 10 wt% (they were calculated using the total weight of polymeric system). It was possible to add up to 10 wt% of waste, while a higher content led to mixing problems.

To obtain a good dispersion, PUA was mixed with either licorice root or palm leaf waste at room temperature for 30 min. Afterwards, the photo-initiator and co-initiator were added. The system was poured in a Teflon mold and left to cure under UV radiation. A curing cycle of 4 h and a post-cure for a further hour at room temperature were performed.

### 2.3. Synthesis of Poly(urethane-acrylate) (PUA)

All glassware was oven dried at 100 °C for more than two hours prior to the synthesis of poly(urethane-acrylate).

In a 25 mL two-necked round bottom flask, 2.5 mL of PEG 400 was heated at 70 °C with 3.6 mg of dibutylbis(lauroyloxy)tin and 3 mL of IPDI for 20 min under nitrogen atmosphere. At the end of the first step, the oligomer was cooled down to 50 °C and then end-capped with HEMA (3 mL), by adding it dropwise to the reaction mixture. The synthesis of UAO was performed at 50 °C for 4 h. Then the reaction was cooled down to room temperature and a specific amount of filler was added and stirred for 30 min at room temperature. Afterwards, benzophenone (120 mg) and methyl diethanolamine (120 mg) were added and the reaction mixture was cast in a Teflon mold and was exposed to the UV-lamp for 10 min in order to obtain PUA.

### 2.4. Chemical Characterization of Waste and Polymer Matrix

The licorice root and palm leaf were milled for 15 min at 20 Hz using a Retzsch MM 400 (Retsch GmbH, Haan, Germany) ball mill for size reduction and homogenization with a 35 mL chamber in stainless steel and equipped with stainless steel grinding balls with diameter of 20 mm.

Fillers and PUA-based composites were characterized by FTIR-ATR analysis, performed with a Perkin-Elmer FT-IR spectrometer Spectrum Two UATR, equipped with ZnSe crystal. The measurements were performed in a 400–4000 cm^−1^ range at a 2 cm^−1^ resolution, 4 scans and processed by a Perkin-Elmer FT-IR Spectrometer Spectrum Two UATR.

Thermogravimetric analysis (TGA) of the two types of waste was performed to evaluate their thermal stability. TGA analysis was carried out using a SetSys Evolution (Setaram Instrumentation, Caluire, France) thermogravimetric analyzer, equipped with Al_2_O_3_ crucibles. 30–35 mg of samples were heated from room temperature to 800 °C, under nitrogen atmosphere with a heating rate of 10 °C/min.

The molecular weights of polymer matrix were evaluated by gel permeation chromatography (GPC). The measurements were carried out with an Agilent 1260 Infinity II Multi Detector Suite (MDS) device. In this device, there was an Agilent 1260 Infinity Quaternary Pump (G7111B), containing a 4-channel vacuum degasser to pump the eluent into the system. The auto sampler was G7129A and the thermostatic column compartment G7116A. The used device consisted of three different detectors (G7800A): a dual light scattering detector (measuring in the angles of 15° and 90°), an RI detector, and a VS-detector. The THF mobile phase contained 250 ppm of BHT (butylhydroxytoluene) and the flow rate was fixed at 1.0 mL/min. In the measurements and data analysis, an Agilent GPC/SEC Software, was used. GPC system was equipped with two columns in series (PLgel MIXED-C and PLgel MIXED-D) and before the columns there was also a guard column (Agilent GPC/SEC Guard Column) kept at a temperature of 35°C. The standards used in the measurements for column calibration were PSs with different Mp values in the range of 580–283,800 g/mol.

The chemical nature of poly(urethane-acrylate) was determined by nuclear magnetic resonance (NMR). ^1^H-NMR and ^13^C-NMR spectra were recorded on a Varian Mercury 400 (400 MHz or 100 MHz, respectively). Chemical shifts are quoted in ppm and are referenced to residual protons in the deuterated solvent as the internal standard. In this case CDCl_3_ (7.26 ppm for ^1^H and 77.0 ppm for ^13^C) was used and the amount of tested sample was 20 mg, placed in a DURAN^®^ borosilicate glass tubing NMR analysis. Splitting patterns are designated as follows: s, singlet; d, doublet; t, triplet; q, quartet; m, multiplet.

### 2.5. Thermal and Mechanical Characterization of Composite Materials

Mechanical properties of PUA-based composite materials were evaluated in tension in accordance with ASTM D3039 with a Zwick/Roell Z010 (Zwick/Roell, Ulm, Germany) universal testing machine equipped with a 1 kN load cell. Tests were carried out in displacement control with a speed of 10 mm/min while the actual deformation was assessed by using a high-resolution sensor arm extensometer with a gauge length of 30 mm. At least three samples were used for each composite formulation. Hardness measurements (Shore A) were performed according to ASTM D 2240, using a Zwick/Roell Hardness Tester. The hardness value for each sample was calculated as the average of 20 measurements.

Thermogravimetric analysis (TGA) of composite materials (30–35 mg in alumina crucibles) was carried out from room temperature up to 800 °C with a heating rate of 10 °C/min in a nitrogen atmosphere with a SetSys Evolution (Setaram Instrumentation, Caluire, France) thermogravimetric analyzer. The thermal behavior of the different formulations was investigated by differential scanning calorimetry (DSC). Samples (9–10 mg in an aluminum concave pan with pierced lid) were analyzed in a DSC 214 Polyma (NETZSCH-Gerätebau GmbH, Selb, Germany), according to the following thermal program: heating from −20 °C to 210 °C (5 min hold), cooling to −20 °C (5 min hold) and heating to 210 °C, all steps at 10 °C/minute. Composite fracture surfaces were investigated by scanning electron microscopy (SEM), using a FE-SEM Mira3 by Tescan (Brno, Czech Republic). Prior to analysis the specimens were sputter coated with gold to prevent charging.

## 3. Results and Discussion

### 3.1. Synthesis of Poly(urethane-acrylate) (PUA)

Poly(urethane-acrylate) synthesis involved three steps: (1) urethane oligomer formation in the presence of dibutyl-tin(dilaurate) (DBTDL), with a molecular weight ranging from 600 to 6000 Da, (2) end-capping agent addition to the NCO-terminated urethane pre-polymer, (3) radical polymerization by UV radiation of vinyl group of end-capped acrylates, in the presence of photo-initiator and co-initiator [18]. The numbers in bold indicate the different chemicals used for synthesis and obtained after that.

**Scheme 1:**
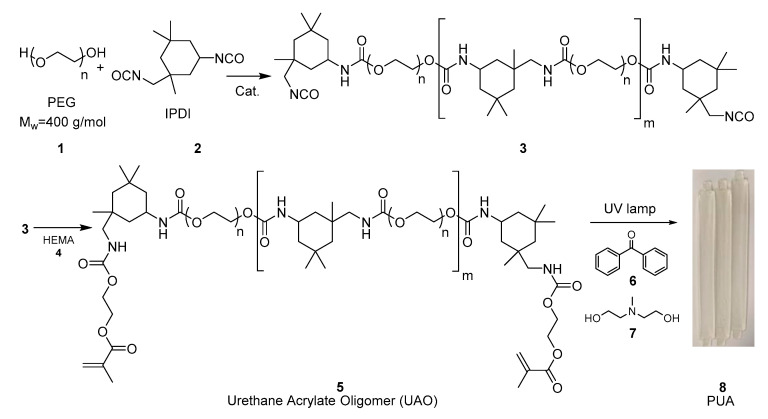
Overall synthesis of PUA formation with samples after curing.

In the first step, a pre-polymer, **3**, was synthetized starting from polyethylene glycol (PEG, **1**) and isophorone diisocyanate (IPDI, **2**, Scheme 1). Dibutyl-tin(dilaurate) (DBTDL), generally employed during this synthesis, allowed to achieve a weight average molecular weight of Mw = 1500 Da [19].

The NCO-terminated urethane pre-polymer obtained, **3**, was involved in the reaction with 2-hydroxyethyl methacrylate (HEMA, **4**) in order to obtain urethane-acrylate oligomer (UAO, **5**, Scheme 1) with a weight average molecular weight of Mw = 1600 Da. Afterwards, the photo-polymerization of **5** in the presence of benzophenone, **6**, as photo-initiator and methyldiethanolamine, **7**, as co-initiator was performed (Scheme 1).

Urethane pre-polymers with different M_w_ were investigated, in order to study the effect of molecular weight on urethane-acrylate oligomer (UAO) formation. Values higher than 2000 Da did not lead to the formation of **5** (entry 1.1, Table 1). In particular, the isocyanate peak remains unaffected after 48 h. This behavior suggests that increasing the M_w_ of NCO urethane pre-polymer makes end-groups less available for further reaction with acrylate compounds. On the other hand, decreasing the molecular weight down to 1500 Da it was possible to obtain UAO with an M_w_ of 1600 Da (entry 1.2, Table 1).

#### Chemical Characterization

**Figure 2:**
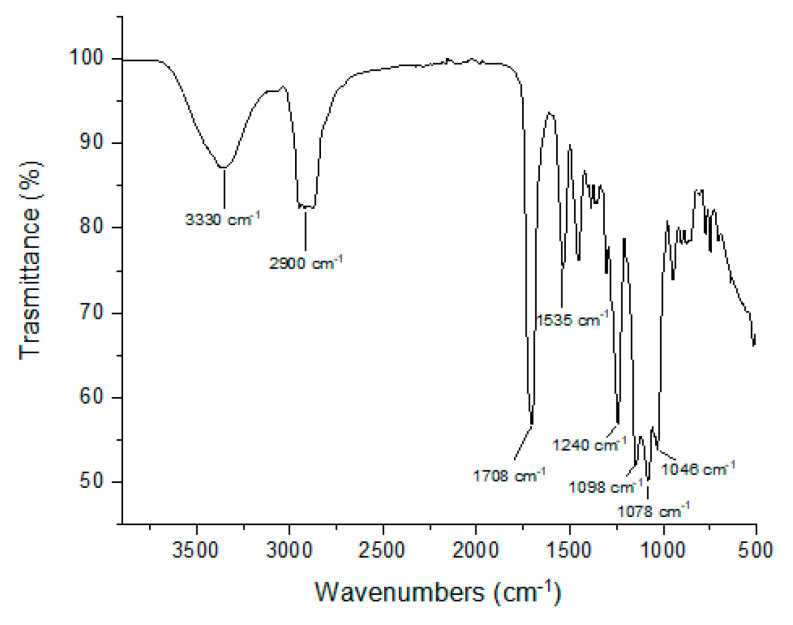
FTIR spectrum of poly(urethane-acrylate) (PUA).

The formation of **3** and **5** was confirmed by FTIR-ATR (Figure S2*)* and NMR analysis (Figure S3*)*, where all the characteristic peaks of NCO-terminated urethane prepolymer and urethane-acrylate oligomer were observed. The final PUA, **8**, was characterized by FTIR analysis, as shown in Figure 2. The FTIR spectrum confirms that the carbon-carbon double bonds are involved in cross-linking reaction by photo-polymerization and the characteristic peaks of acrylate double bond (1634 cm^−1^ and 814 cm^−1^) are not detected [20].

The characteristic absorption peaks at 3330 cm^−1^ and 1535 cm^−1^ were attributed to the stretching vibration absorption of N–H in the urethane group, and the absorption bands in the range of 2800–2930 cm^−1^ were related to the stretching vibration of C–H. The peaks at 1708 cm^−1^ and 1240 cm^−1^ were related to C=O and C–N stretching vibration, respectively. The absorption bands in the range of 1046–1098 cm^−1^ were attributed to C–O stretching vibration.

### 3.2. Characterization of Licorice Root and Palm Leaf Wastes

**Figure 3:**
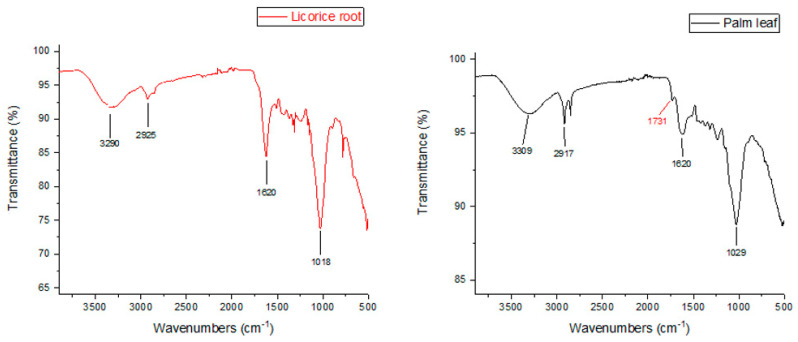
FTIR spectra of licorice root and palm leaf.

Licorice root and palm leaf wastes were characterized by FTIR-ATR, TGA and SEM analysis. The FTIR spectra show a very strong peak around 3300 cm^−1^, related to the –OH stretching, as well as the aliphatic –CH and –CH_2_ stretching at 2856–2925 cm^−1^. The peaks at around 1620 cm^−1^ and 781 cm^-1^ are assigned to aromatic skeletal vibration allied to the out-of-plane stretching and bending of aromatic –CH, respectively. In contrast, those in the region from 1031 to 1160 cm^−1^ are assigned to the –C–O stretching vibration, and the peak at 1427 cm^−1^ is assigned to the –CH, –CH_2_ bending of aliphatic carbons [15]. Palm leaf shows also the –C=O stretching vibration at around 1731 cm^−1^ (Figure 3). This peak is related to ester bond of carboxyl group of ferulic and *p*-coumaric of lignin or acetyl ester groups and uronic groups of hemicelluloses [21].

**Figure 4:**
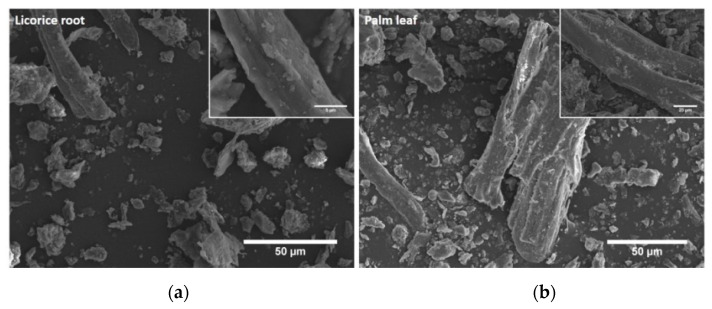
SEM micrographs of milled licorice root (**a**) inset scale bar = 5 µm and palm leaf (**b**) inset scale bare = 20 µm fillers.

From SEM analysis, the natural fillers after the milling process exhibited irregular shapes and significant geometrical variability, with varying aspect ratios, as shown in Figure 4. In particular, both fillers displayed a morphology far from the fibrous one. From SEM micrographs, the length and diameter of 60 particle-like fillers for palm leaf and licorice root were measured to determine the average aspect ratio (length/diameter). In both cases the aspect ratio, on average, was lower than 5, thus suggesting a non-optimized reinforcement effect over the neat PUA, as predicted by Halpin-Tsai model and by the lower filler/matrix interfacial area. Licorice root filler was characterized by a more refined structure compared to palm leaf waste.

**Figure 5:**
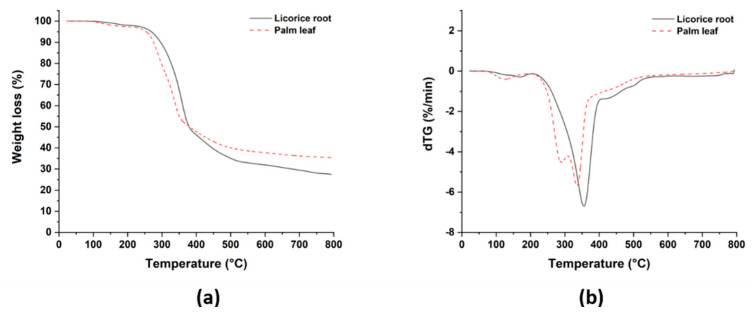
Thermal degradation behavior of palm leaf and licorice root wastes: (**a**) weight loss vs. temperature and (**b**) derivative of weight loss (percent/minute) vs. temperature.

The thermal degradation behavior was also investigated through TGA and the resulting thermograms and derivative thermograms are reported in Figure 5. The initial mass loss up to 200 °C was ascribed to moisture evaporation, which is quite common in natural fibers [22], albeit it was higher in palm leaf compared to licorice root. Thereafter, the onset of thermal degradation, corresponding to 5 wt% loss, occurred at 270 °C for licorice root, about 15 °C higher than palm leaf. The better thermal stability of licorice root was confirmed also with increasing temperature, with a maximum degradation temperature at 355 °C (Table 2). All these values are in line with those exhibited by other common natural fibers [23].

It is also worth mentioning that palm leaf fillers, in contrast to licorice root and other natural fibers, featured two well distinct degradation peaks in the range 250–350 °C: a first peak at 290 °C and a second peak at 335 °C. These peaks can be ascribed to the thermal decomposition of hemicellulose and cellulose in an inert atmosphere, respectively [24]. In contrast, the low-temperature peak was overlapped to the cellulose main peak and consequently it was not obvious in the case of licorice root. The high-temperature tail shown in both curves of Figure 5 is due to the degradation of lignin [25], which resulted in a higher charred organic material residue for palm leaf filler.

To determine the content of cellulose, hemicellulose and lignin in palm leaf and licorice root a chemical composition analysis was performed. To this purpose, selective extraction methods were used. Lignin was isolated from biomass through dissolving method with deep eutectic solvent (acetic acid: choline chloride, 2:1) [26]. The solid residue was separated by centrifugation, and then washed to obtain cellulose. Hemicellulose was isolated by an alkaline extraction method [27].

The chemical composition analysis of natural fillers indicates that the content of lignin in palm leaf is higher than licorice root, while the content of cellulose is lower. The amount of hemicellulose is the same for both biomasses (Table 3).

### 3.3. Thermal and Mechanical Characterization of PUA Composites

**Figure 6:**
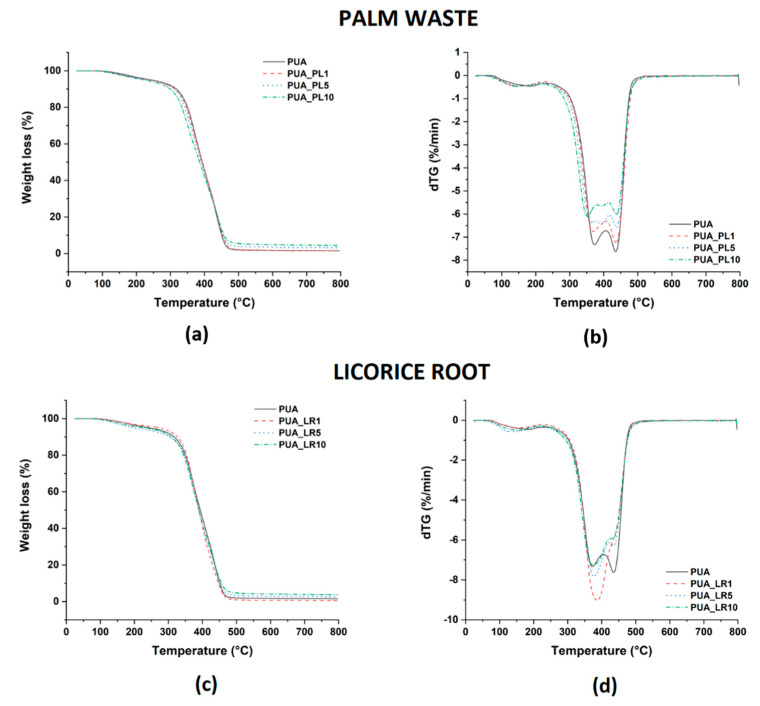
Thermal degradation behavior of palm leaf and licorice root reinforced composites: (**a**,**c**) weight loss percent vs. temperature and (**b**,**d**) derivative of weight (percent/minute) (dTG) vs. temperature.

TGA analysis shows that both fillers, when added in the PUA matrix, caused a reduction in the thermal stability of the resulting composites compared to the neat matrix, with palm leaf fillers that performed better compared to licorice waste, as it can be seen in Figure 6. Neat PUA exhibited two main stages of degradation with peak maxima around 370 °C and 430 °C. The weight loss at temperatures lower than 200 °C can be ascribed to the removal of solvent and moisture [28]. The first decomposition region corresponds to the breakage of urethane linkage, i.e., hard segments, while the soft segments are degraded during the second stage. When comparing the carbon-carbon single bonds of the soft segments, the polar groups of the hard segments are less thermally stable and can be more easily decomposed to form an alcohol and isocyanate group [29,30].

As shown in Figure 6, the global decomposition path is not significantly affected by the presence of the fillers, albeit a modest reduction in the temperatures of 10% weight loss and maximum degradation can be observed (Table 4), quite limited in the case of palm leaf filler. In the case of licorice root waste, the fluctuating results can be ascribed to issues related to its dispersion and agglomeration, especially at increasing weight fractions, which could have changed the decomposition diffusion pathway of PUA, affecting the thermal stability.

**Figure 7:**
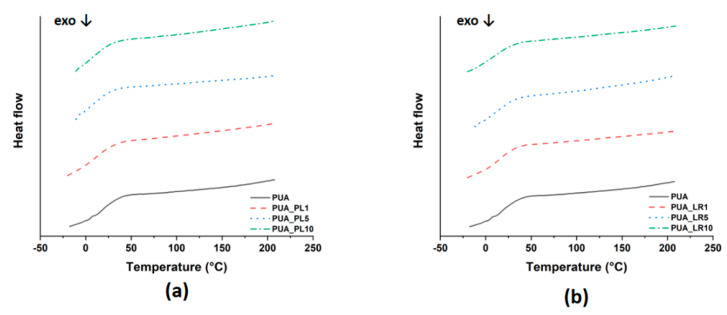
DSC thermograms of the PUA-based composite materials reinforced with: (**a**) palm leaf and (**b**) licorice root.

From TGA results, the presence of fillers did not affect in a significant way the thermal stability of the matrix, and this was confirmed also by DSC analysis. Figure 7 shows the DSC thermograms for neat PUA and all the composite formulations.

DSC thermograms of the PUA-based composites are represented by the second heating cycle and as represented in Figure 7 and Table 4, the glass transition temperature (T_g_) of the PUA composites ranged from 22.8 to 25.7 °C, driven by the hard segment. The glass transition is usually related to the segmental mobility of polymer chains [31], and two major contributions are represented by the physical confinement and possible strong interactions at the polymer chain/filler surface. The presence of natural fillers may restrict the segmental motion of polymer chains close to the filler surface, but the DSC results pointed out no significant changes in T_g_ value after the incorporation of palm leaf or licorice root. A possible reason is the lack of a sound interfacial adhesion between the PUA matrix and both fillers, as will be confirmed by SEM analysis.

**Figure 8:**
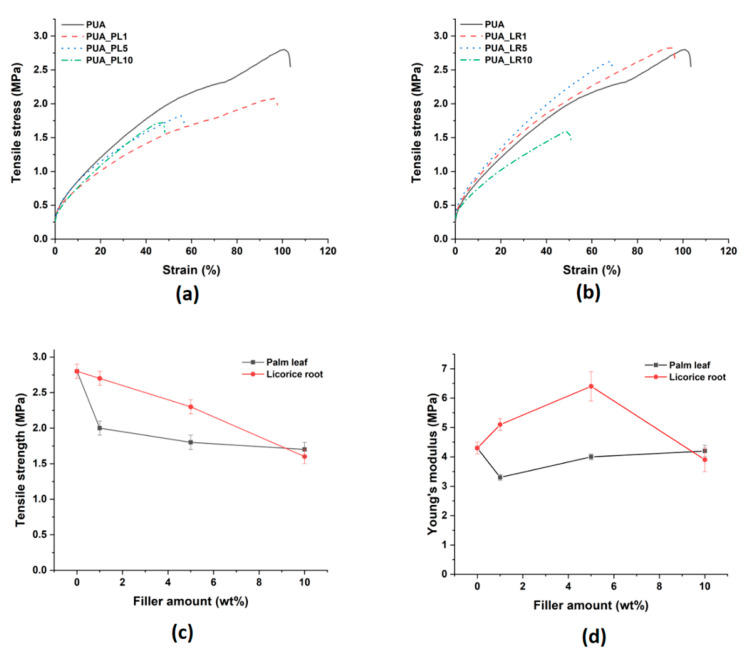
Tensile response of PUA and related composite formulations: (**a**) typical tensile stress vs. strain curves for PUA-palm leaf composites; (**b**) typical tensile stress vs. strain curves for PUA-licorice root composites; (**c**) tensile strength as a function of filler amount; (**d**) Young’s modulus as a function of filler amount.

The low glass transition temperature resulted in composite materials showing a high ductility, only partially impaired by increasing amount of filler, and poor stiffness, as evaluated from tensile tests, whose results are summarized in Figure 8. The tensile strength of the matrix was not improved by the incorporation of both fillers, while Young’s modulus showed a different behavior depending on the type of filler. As a general result, licorice root waste provided the best mechanical properties of the resulting composites, and at the expense of a 17% reduction in tensile strength an increase in stiffness by 50% can be achieved with a filler amount of only 5 wt%, while still retaining an elongation at break higher than 60%. The improved performance offered by licorice root can be explained by considering the higher amount of cellulose, which is the chemical constituent governing the mechanical performance of natural fibers [22]. The lack of a strong interfacial adhesion prevented the composites from achieving higher mechanical properties, especially in terms of tensile strength. In Figures S4 and S5 it is possible to observe the fracture surfaces for the composite formulations as a function of filler amount. Compared to the neat polymer, a roughening of the fracture surface is clearly visible with increasing filler content, irrespective of filler type, ascribed to the complex interactions of the propagating cracks with the fillers. However, fillers highlighted a poor interfacial adhesion with the PUA matrix supported by the extensive presence of filler pull-out and debonding, with clear gaps at the interface with the polymer, which limited the tensile strength that could be achieved. In particular, for composites reinforced with licorice root waste, it is possible to observe the presence of agglomerations and clusters at the highest weight fraction which led to a severe reduction in tensile strength and modulus.

**Figure 9:**
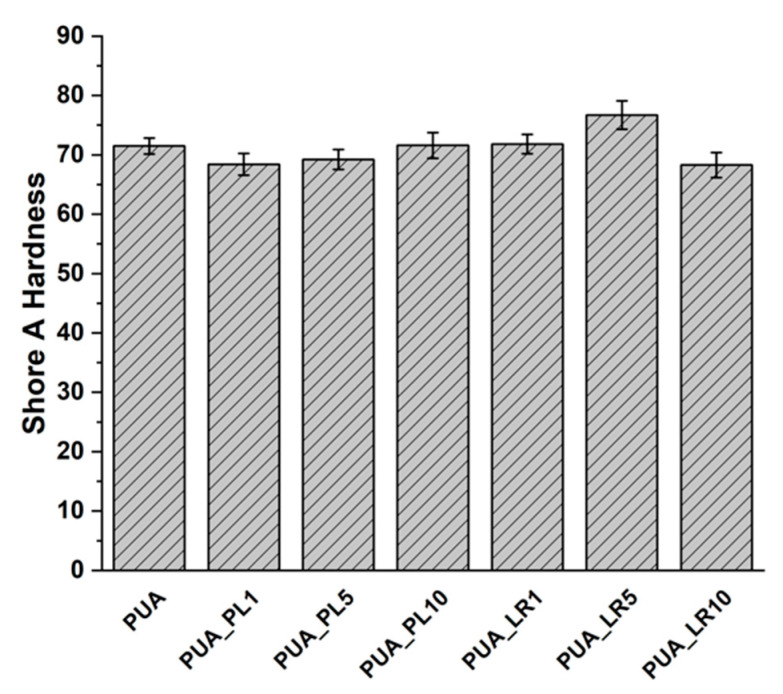
Shore A hardness data of PUA and related composite formulations.

A summary of hardness data is presented in Figure 9. Results clearly show that the hardness of composites is found to slightly increase with increasing filler content for both PUA-palm leaf and PUA-licorice root composites. As already observed for tensile properties, in particular, for tensile stiffness, different fillers resulted in a different global behavior. Licorice root waste provided an increase in composite hardness compared to neat PUA up to 5 wt%, because at higher loading its inhomogeneous dispersion led to a decreased hardness value. On the contrary, palm leaf filler was not able to increase the hardness of the matrix which plateaued around 70.

The introduction of waste natural fillers in PUA matrices needs to be optimized mainly in terms of interfacial adhesion, but it seems a viable and promising route for upcycling agricultural wastes as the mechanical properties achieved compare favorably with those reported in other studies where PUA matrices have been traditionally reinforced with inorganic nanoparticles, such as graphene oxide [32], silica [33,34], indium tin oxide [35] and barium titanate [36].

## 4. Conclusions

In conclusion, we successfully developed an efficient, simple, practical approach for the manufacture of a poly(urethane-acrylate) composite, including either of two powdered lignocellulosic wastes, namely licorice root and palm leaf, in an amount of up to 10%. This methodology allows a wide substrate scope, utilizes readily available chemical and lignocellulosic materials, and provides operational simplicity. The composite showed a promising increase in hardness with respect to the pure polymer, with no substantial degradation of thermal stability and an expected yet acceptable reduction of tensile strength in combination with an increase in stiffness for licorice-PUA composites. Interfacial adhesion between the polymer and the filler would need some improvement in the future in view of the possible introduction of higher amount of waste, which will further improve the hardness and stiffness of the composite, allowing poly(urethane-acrylate) to act as an even more effective coating material. In general terms, efforts to develop more direct applications in the chemical community are in progress in our laboratory.

## Figures and Tables

**Table 1:** Effect of molecular weight on UAO formation.

Entry	M_n_^a^ (g/mol)	M_w_^a^ (g/mol)	M_n_^b^ (g/mol)	M_w_^b^ (g/mol)	Conv.^c^ (%)
1.1	2500	4000	-	-	-
1.2	1000	1500	1200	1600	84

^a^ GPC analysis of NCO-terminated urethane pre-polymer (detector RI, refractive index). ^b^ GPC analysis of urethane-acrylate oligomer (detector RI, refractive index).^c^ Determined by GPC analysis, taking into account the ratio between the areas of the peaks relative to the HEMA (b, Figure S1) and to the oligomer (a, Figure S1).

**Table 2:** Thermal degradation parameters for both natural fillers.

Specimen	T_d5_ (°C) ^a^	T_d10_ (°C) ^b^	T_max_ (°C) ^c^
Palm leaf	254.8 ± 1.8	275.2 ± 1.3	335.2 ± 2.2
Licorice root	269.9 ± 2.0	295.8 ± 0.9	354.7 ± 2.5

^a^ Temperature at 5% weight loss, ^b^ temperature at 10% weight loss, ^c^ temperature at maximum degradation rate.

**Table 3:** Amounts of cellulose, lignin, and hemicellulose in the two types of biomass waste.

Specimen	Cellulose ^a^ (%)	Lignin ^a^ (%)	Hemicellulose ^a^ (%)	Total Cont. ^b^ (%)
Palm leaf	26	30	12	68
Licorice root	44	12	15	71

^a^ All% are expressed as enriched fractions. ^b^ The remaining components of biomass are extractives and salts.

**Table 4:** Thermal behavior of the PUA-based composites.

Specimen	T_d5_ (°C) ^a^	T_d10_ (°C) ^b^	T_max_ (°C) ^c^	T_g_ (°C) ^d^
PUA	237.9 ± 5.1	317.6 ± 1.9	434.7 ± 1.9	26.3 ± 0.6
PUA_LR1	261.4 ± 9.5	325.0 ± 1.6	391.9 ± 4.4	24.1 ± 0.4
PUA_LR5	202.7 ± 5.3	303.5 ± 1.3	377.7 ± 1.2	22.8 ± 0.7
PUA_LR10	222.2 ± 5.2	306.4 ± 2.4	373.0 ± 1.5	23.2 ± 0.6
PUA_PL1	234.8 ± 2.7	310.9 ± 2.6	433.1 ± 2.7	24.7 ± 0.6
PUA_PL5	236.3 ± 2.6	307.9 ± 2.0	434.5 ± 2.4	25.4 ± 0.5
PUA_PL10	223.5 ± 1.9	298.0 ± 3.2	423.6 ± 1.7	25.7 ± 0.7

^a^ Degradation temperatures at 5% weight loss, ^b^ degradation temperatures at 10% weight loss, ^c^ maximum degradation rate, ^d^ glass transition temperature obtained by DSC (2nd heating).

## Data Availability

The data presented in this study are available in this article and in the supplementary information.

## Supplementary Materials

The following are available online, Figure S1: GPC chromatogram of UAO (entry 1.2), Figure S2: FTIR spectrum of compounds **3** and **5**, Figure S3: NMR spectrum of compounds **3** and **5**, Figure S4: SEM micrographs detailing the fracture surfaces of PUA composites reinforced with licorice root waste, Figure S5: SEM micrographs detailing the fracture surfaces of PUA composites reinforced with palm leaf waste.

## Sample Availability

Samples of the compounds are available from the authors.
