# Supplementary material for: Chemical and Mechanical Characterization of Licorice Root and Palm Leaf Waste Incorporated into Poly(urethane-acrylate) (PUA)
Source: Molecules. 2021 Dec 19;26(24):7682. doi: 10.3390/molecules26247682 (PMC8705998; doi:10.3390/molecules26247682)
Supplement: Supplementary file 1 [file molecules-26-07682-s001.zip › molecules-1494309-supplementary.pdf]

Supporting Information

Chemical and Mechanical Characterization of Licorice Root and Palm Leaf Waste Incorporated into Poly(Urethane-Acrylate) (PUA)

Serena Gabrielli [1,*], Genny Pastore [1], Francesca Stella [1], Enrico Marcantoni [1], Fabrizio Sarasini [2], Jacopo Tirillò [2] and Carlo Santulli [3,*]

[1] ChIP Building, School of Science and Technology, Università degli Studi di Camerino, via Madonna delle Carceri, 62032 Camerino, Italy; genny.pastore@unicam.it (G.P.); francesca.stella@unicam.it (F.S.); enrico.marcantoni@unicam.it (E.M.)
[2] Department of Chemical Engineering Materials Environment, Sapienza-Università di Roma, Via Eudossiana 18, 00184 Roma, Italy; fabrizio.sarasini@uniroma1.it (F.S.); jacopo.tirillo@uniroma1.it (J.T.)
[3] Geology Section, School of Science and Technology, Università degli Studi di Camerino, via Gentile III da Varano 7, 62032 Camerino, Italy
* Correspondence: serena.gabrielli@unicam.it (S.G.); carlo.santulli@unicam.it (C.S.)

Table of Contents

Figure S1. GPC chromatogram of UAO (entry 1.2)………………………………………………………………………………… 2

Figure S2. FTIR spectrum of compounds **3** and **5**……………………………………………………………………….. 2

Figure S3. NMR spectrum of compounds **3** and **5**……………………………………………………………………………… 3

Figure S4. SEM micrographs detailing the fracture surfaces of PUA composites reinforced with licorice root waste… 6

Figure S5. SEM micrographs detailing the fracture surfaces of PUA composites reinforced with palm leaf waste…… 7

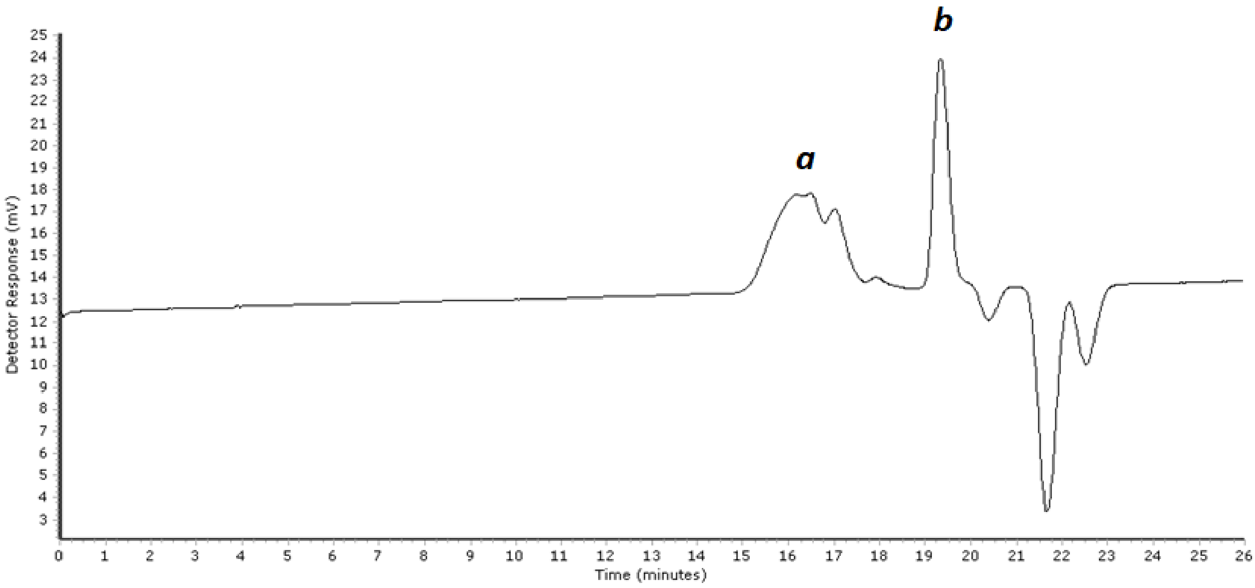

Figure S1. GPC chromatogram of UAO (entry 1.2).

During the analysis, the GPC column separates the polymer molecules based on their size in solution, and the detector determines the amount of material eluting from the column as a function of retention time. This is converted to molecular weight using a calibration curve – a graph that relates retention time (time of elution from the column) to the molecular weight of the polymer. The calibration curve is itself generated by the elution behavior of a series of polymer standards of known molecular weight. During the calculations the peak for the sample eluting from the column is divided into 'slices', and the retention time of each slice is used to determine its molecular weight by reference to the calibration curve. The area or height of the slice allows the percentage of the whole sample in the slice to be determined, and from the percentage and molecular weight data the molecular weight distribution is calculated.

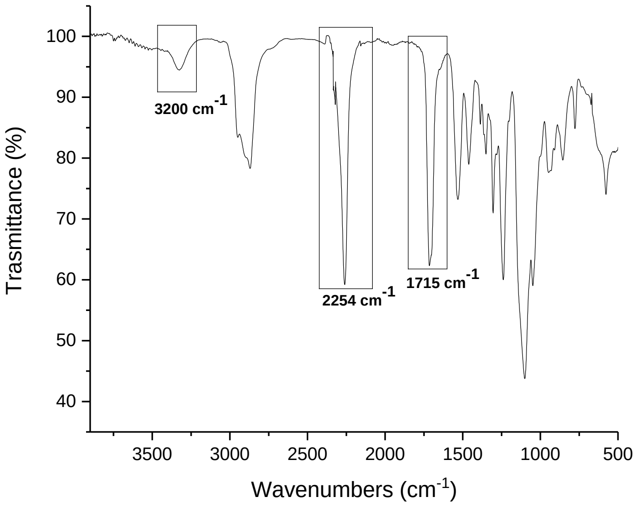

FTIR spectrum of **3**.

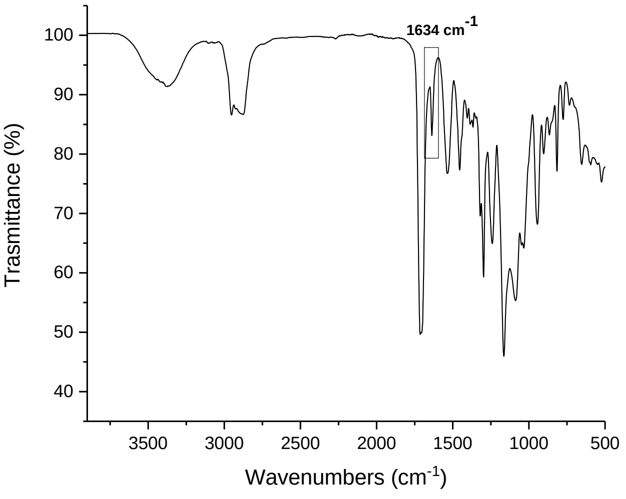

FTIR spectrum of **5**.

Figure S2. FTIR spectrum of compounds **3** and **5**.

The FTIR spectrum of compound **3** shows the characteristic peaks of NCO end-groups at 2254 cm^{-1}, C=O of the urethane bond at 1715 cm^{-1} and the stretching of -NHR functionalities of the urethane group at 3200 cm^{-1}.
The FTIR spectrum of compound **5** shows the characteristic peaks of acrylate double bond at 1634 cm^{-1} (C=C stretching) and the disappearance of isocyanate peak at 2254 cm^{-1} confirms the formation of UAO, due to the reaction between NCO groups with HEMA.

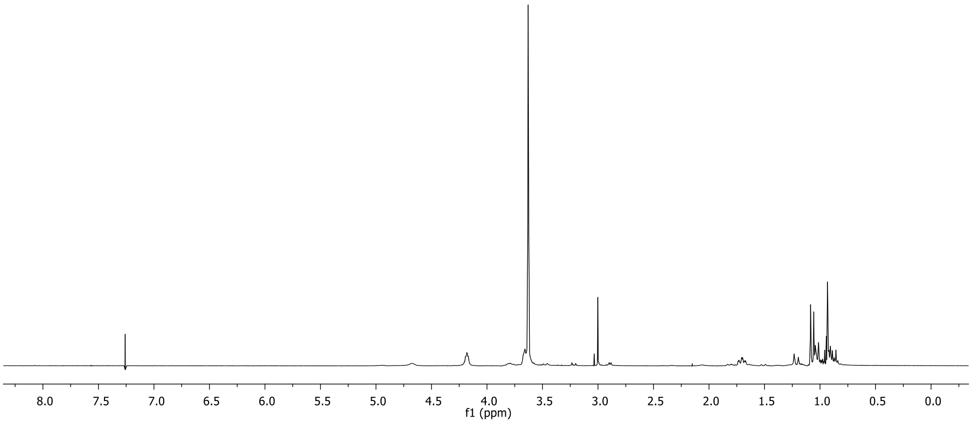

^{1}H-NMR of compound **3**.

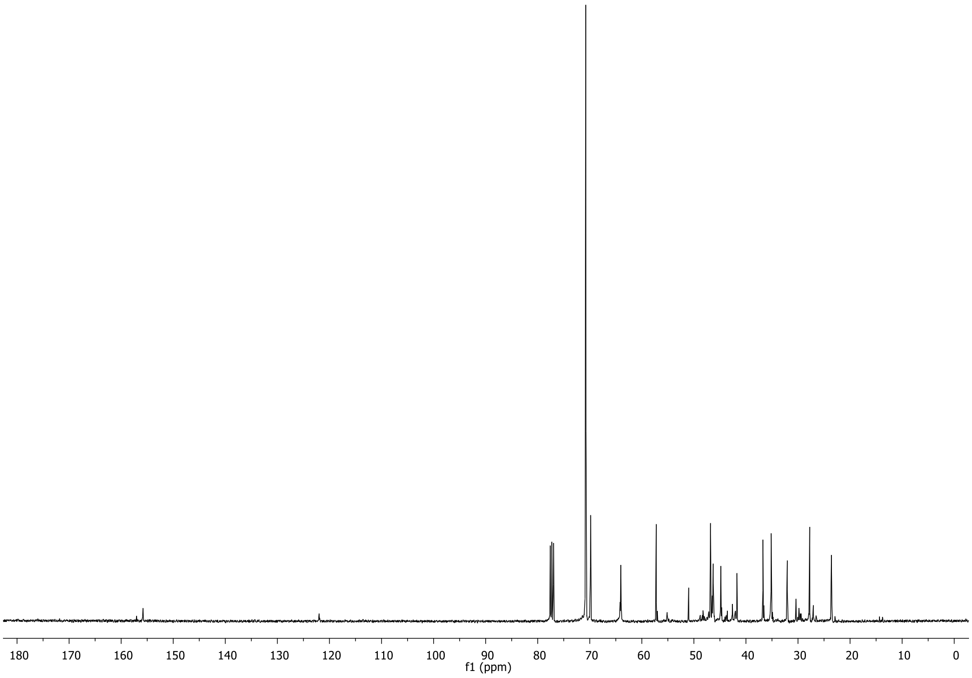

^{13}C-NMR of compound **3**.

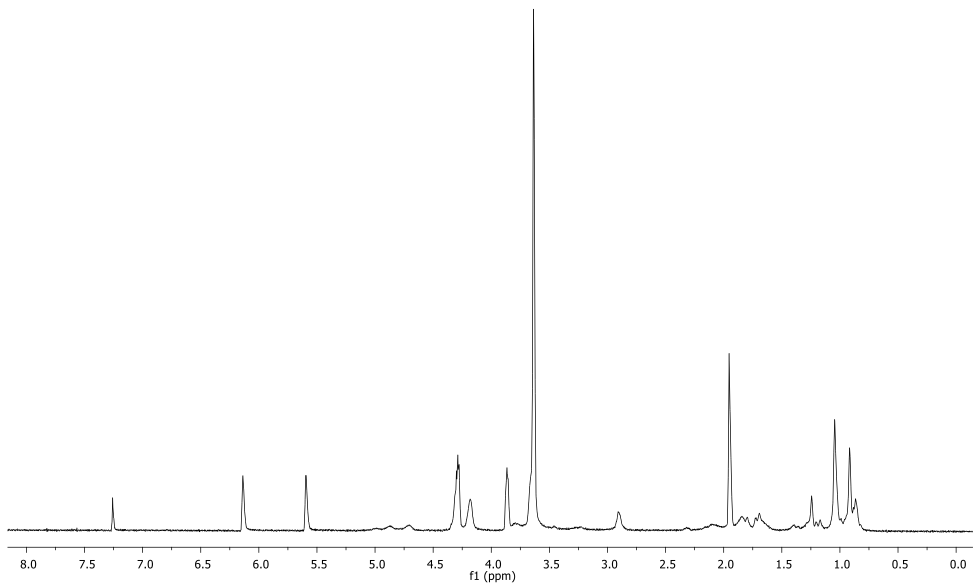

^{1}H-NMR of compound **5**.

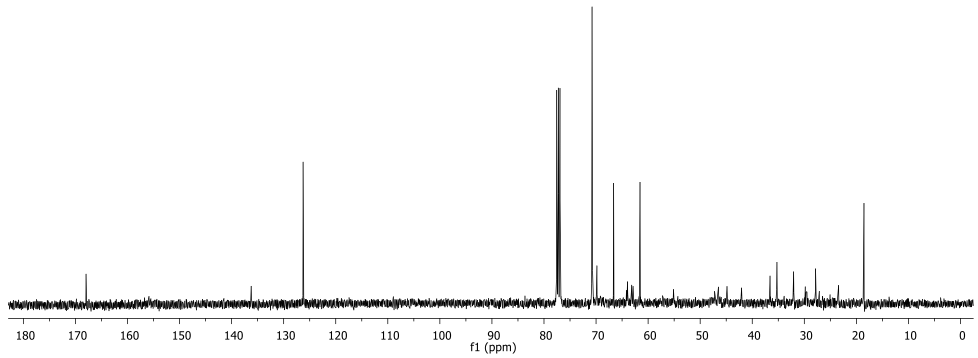

^{13}C-NMR of compound **5**.

Figure S3. NMR spectrum of compounds **3** and **5**.

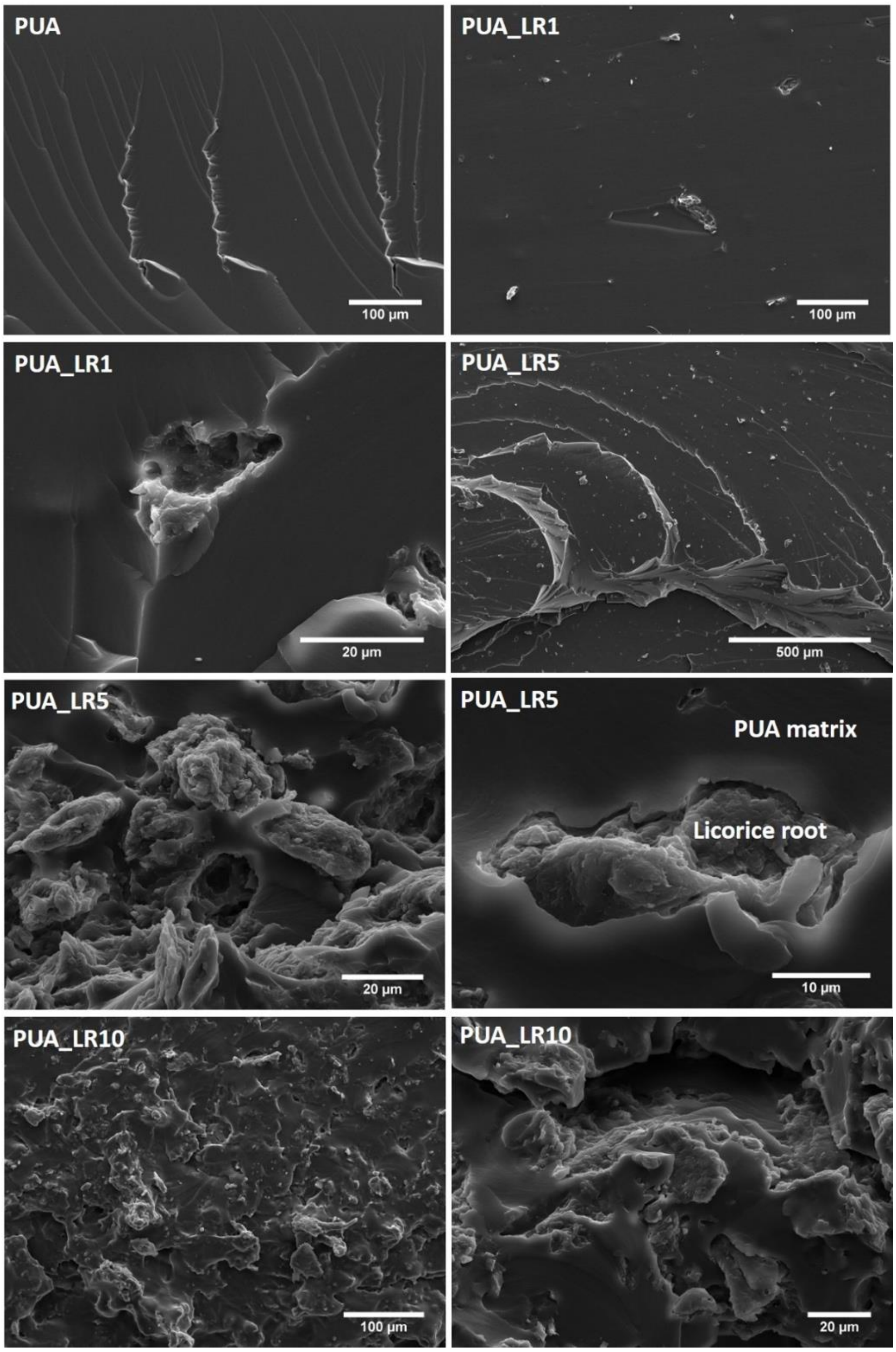

Figure S4. SEM micrographs detailing the fracture surfaces of PUA composites reinforced with licorice root waste.

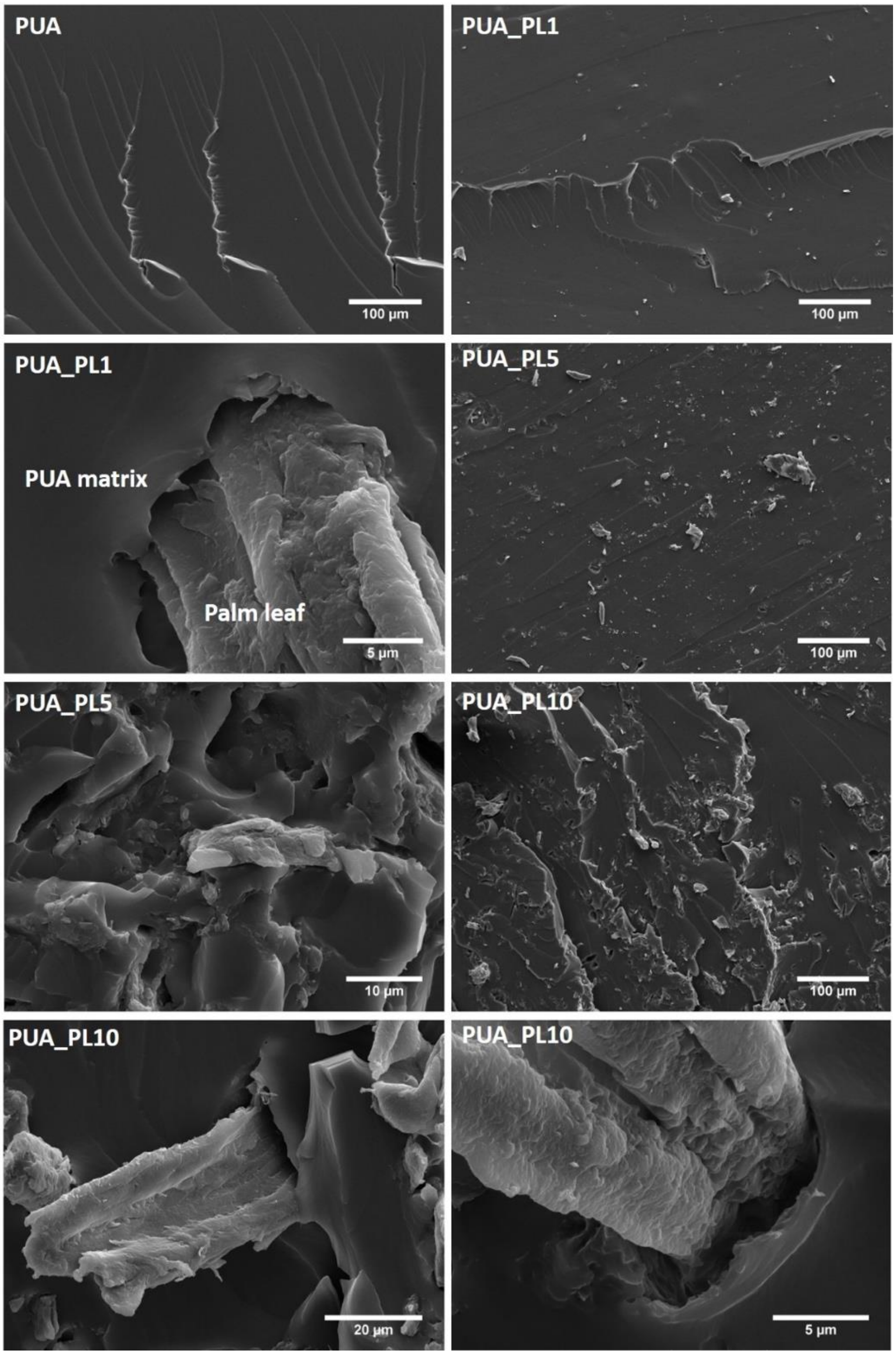

Figure S5. SEM micrographs detailing the fracture surfaces of PUA composites reinforced with palm leaf waste.
